# EnGCI: enhancing GPCR-compound interaction prediction via large molecular models and KAN network

**DOI:** 10.1186/s12915-025-02238-3

**Published:** 2025-05-15

**Authors:** Weihao Liu, Xiaoli Li, Bo Hang, Pu Wang

**Affiliations:** Computer School, Hubei University of Arts and Science, Longzhong Road, Xiangyang, 441053 Hubei China

**Keywords:** GPCR-compound interaction prediction, Large molecular models, Kolmogorov-Arnold network, Graph isomorphism network

## Abstract

**Background:**

Identifying GPCR-compound interactions (GCI) plays a significant role in drug discovery and chemogenomics. Machine learning, particularly deep learning, has become increasingly influential in this domain. Large molecular models, due to their ability to capture detailed structural and functional information, have shown promise in enhancing the predictive accuracy of downstream tasks. Consequently, exploring the performance of these models in GCI prediction, as well as evaluating their effectiveness when integrated with other deep learning models, has emerged as a compelling research area. This paper aims to investigate these challenges.

**Results:**

This study introduces EnGCI, a novel model comprising two distinct modules. The MSBM integrates a graph isomorphism network (GIN) and a convolutional neural network (CNN) to extract features from GPCRs and compounds, respectively. These features are then processed by a Kolmogorov-Arnold network (KAN) for decision-making. The LMMBM utilizes two large-scale pre-trained models to extract features from compounds and GPCRs, and subsequently, KAN is again employed for decision-making. Each module leverages different sources of multimodal information, and their fusion enhances the overall accuracy of GPCR-compound interaction (GCI) prediction. Evaluating the EnGCI model on a rigorously curated GCI dataset, we achieved an AUC of approximately 0.89, significantly outperforming current state-of-the-art benchmark models.

**Conclusions:**

The EnGCI model integrates two complementary modules: one that learns molecular features from scratch for the GPCR-compound interaction (GCI) prediction task, and another that extracts molecular features using pre-trained large molecular models. After further processing and integration, these multimodal information sources enable a more profound exploration and understanding of the complex interaction relationships between GPCRs and compounds. The EnGCI model offers a robust and efficient framework that enhances GCI predictive capabilities and has the potential to significantly contribute to GPCR drug discovery.

## Background

G protein-coupled receptors (GPCRs) are the largest family of membrane proteins in the human genome, responsible for mediating physiological responses to hormones, neurotransmitters, and environmental stimuli [1]. Due to their pivotal roles in various biological processes, GPCRs have become one of the most prominent drug targets [2, 3]. A study by Hauser indicates that approximately 34% of US Food and Drug Administration (FDA)-approved drugs (475 in total) target 108 different GPCRs [4]. However, only a small fraction of the GPCR family has been thoroughly explored for drug discovery, leaving significant potential for the identification of new therapeutic targets [5].

The development of GPCR-targeted drugs requires a comprehensive understanding of their three-dimensional structures to optimize drug-receptor interactions and enhance drug selectivity and efficacy [6–9]. Techniques such as X-ray crystallography and cryo-electron microscopy have been crucial in resolving the 3D structures of GPCRs, providing more precise molecular targets for drug design. Upon ligand binding, GPCRs undergo conformational changes that activate intracellular G proteins and initiate signaling. Understanding these conformational changes is essential for drug design, particularly in elucidating how ligands bind to GPCRs and trigger receptor activation [10]. However, due to the limited availability of high-resolution structural data and the high costs associated with these techniques, researchers are increasingly turning to computational methods to predict GPCR-ligand interactions [11].

With advances in computational biology, predictive models based on molecular dynamics simulations and ligand docking are emerging as powerful tools for studying GPCRs. These methods allow researchers to identify key binding sites and simulate GPCR-ligand interactions in a computational environment, complementing experimental data. However, molecular dynamics and docking approaches still face challenges in capturing the complex behavior of GPCRs, especially when dealing with highly flexible or unknown structures [12, 13]. To address these challenges, machine learning (ML) and deep learning (DL) techniques have been applied to GPCR-ligand interaction prediction [14, 15]. Early machine learning models, such as support vector machines and random forests, performed well in predicting GPCR-ligand interactions based on sequence and structural features [16, 17]. For example, Qiu et al. [18] proposed a BOW-GBDT model that combines gradient boosting decision trees (GBDT) with artificial neural networks (ANN) to improve prediction accuracy. The model extracts features from GPCR sequences using a bag-of-words (BOW) approach and from drugs using discrete wavelet transform (DWT). To address the challenge of imbalanced datasets, the SMOTE algorithm is employed, achieving superior accuracy and generalization compared to traditional models. Karimi et al. [19] developed a machine learning-based model for predicting GPCR-ligand interactions, incorporating multiple sequence features to significantly improve prediction accuracy. However, these models rely on manual feature engineering, limiting their scalability and generalization abilities [20–23].

In recent years, deep learning techniques, particularly deep neural networks (DNNs), convolutional neural networks (CNNs), and recurrent neural networks (RNNs), have made significant advancements in GPCR prediction research [24, 25]. These models excel in capturing complex feature representations from large-scale biological data [26, 27]. DNNs and CNNs have been employed to predict GPCR-ligand interactions, binding affinities, and even off-target drug effects with greater accuracy than traditional methods [28, 29]. For instance, Chen et al. [30] proposed TransformerCPI, a model designed for predicting compound-protein interactions (CPI) using the transformer architecture. Unlike traditional methods that rely on 3D structural data, TransformerCPI uses only sequence-based information from proteins and compounds, making it particularly effective for large-scale predictions when 3D structures are unavailable. By leveraging the self-attention mechanism of transformers, the model captures complex relationships between proteins and compounds. Label-reversal experiments further reduce ligand bias, ensuring the model learns genuine interaction features. This approach significantly improves CPI prediction and is especially useful for high-throughput drug screening and target discovery.

The introduction of graph neural networks (GNNs) has further enhanced GPCR-ligand interaction prediction by allowing researchers to incorporate structural information from molecular graphs into the analysis [31, 32]. Nguyen designed a novel deep learning model called GraphDTA [33], which represents drug molecules as molecular graphs and employs a graph neural network to predict drug-target affinity. Unlike traditional methods that represent drugs as linear sequences, GraphDTA captures the interactions between atoms within a molecule through its graphical representation, retaining richer structural information. GraphDTA combines the strengths of GNNs and CNNs. In this model, drug molecules are represented as molecular graphs and processed by graph convolutional networks (GCNs), graph attention networks (GATs), or similar models. Protein sequences are subjected to feature extraction by a one-dimensional convolutional network, and the resulting representation vectors are used to predict drug-target binding affinity via a fully connected layer, significantly improving prediction accuracy. Building on GraphDTA’s foundational work in drug-target interaction (DTI) prediction, DeepGPCR [34] adapted similar graph-based techniques for GPCR-ligand interaction prediction. While GraphDTA initially focused on drug-target interactions (DTIs), DeepGPCR applies GNNs to capture the spatial and physicochemical properties of GPCR binding pockets and ligands, treating both as molecular graphs. This adaptation allows the model to preserve detailed molecular interactions essential for GPCR-ligand prediction, extending the utility of graph-based DTI models to GPCR-targeted drug discovery.

Multimodal approaches that integrate multiple biological, chemical, and structural data sources are now at the forefront of GPCR-ligand prediction research. These models combine diverse data sources to learn richer feature representations, thereby enhancing predictive performance [35, 36]. The MFD-GDrug model [37], which uses a deep-learning multimodal feature-fusion approach for GPCR-drug interaction (GDI) prediction, has demonstrated significant improvements. In its integration of multimodal data, MFD-GDrug employs evolutionary scale modeling (ESM) for GPCR molecule representation and mol2vec to encode compound molecules. However, mol2vec, a word2vec-inspired molecular representation method, is trained on a relatively small dataset, which limits its capacity for representation. Furthermore, MFD-GDrug employs a feature-level fusion strategy that concatenates features from pre-trained models with other features for downstream decision-making. This approach may weaken the ability to capture the unique properties of individual feature sources, potentially hindering the development of more refined, personalized models.

While previous methods, including molecular dynamics simulations, docking techniques, and earlier machine learning models, have been instrumental in predicting GPCR-ligand interactions, several challenges persist. These include reliance on manual feature engineering, limited ability to capture the complex flexibility of GPCRs, dependence on high-quality 3D structural data, and difficulties in integrating multimodal data effectively. These limitations hinder the scalability and accuracy of existing models, particularly in scenarios where 3D structural information is unavailable or when complex interaction dynamics need to be considered.

Recent advances in large molecular models, which are pre-trained on extensive datasets and capable of learning from both sequence and structural data, have shown significant promise in overcoming these challenges. Models such as Uni-Mol and ESM [38–40] leverage deep learning techniques to capture complex molecular interactions and functional properties, enabling more accurate predictions even in the absence of high-resolution structural data.

To address the challenges outlined above, we propose EnGCI, a novel ensemble model designed to predict GPCR-compound interactions. EnGCI incorporates two complementary modules.

Molecular structure-based module (MSBM): This module integrates GIN and CNN technologies to extract features of compounds and GPCR molecules. These technologies, as demonstrated by numerous previous studies, have exhibited remarkable efficacy in constructing molecular representations and recognizing interaction patterns. Subsequently, these meticulously extracted features are fed into the KAN for further identification of interaction relationships.

Large molecular models-based module (LMMBM): This module takes a different approach by introducing two advanced Transformer-based large molecular models, Uni-Mol and ESM, for feature extraction from compounds and GPCRs, respectively. Both models have been pre-trained on large datasets that cover molecular sequence data, spatial structures, and functional properties, granting them powerful representation learning capabilities. These large models have demonstrated exceptional performance across various downstream tasks. After specific post-processing procedures, the features extracted by these models are sent to KAN for in-depth analysis of interaction relationships.

It is noteworthy that these two modules leverage different multimodal information sources to model the complex relationships between compounds and GPCRs, creating a beneficial complementarity. Finally, by integrating a multi-layer perceptron (MLP), which intelligently learns and fuses the outputs of both modules, we achieve a synergistic and efficient integrated model. This design not only enhances the overall performance of the model but also ensures a comprehensive and precise analysis of GPCR-compound interaction relationships.

The primary contributions of our work can be summarized as follows:

- We have proposed a novel ensemble model for GCI prediction and achieved optimal predictive performance on a rigorously constructed GPCR-compound interaction dataset.
- We have incorporated cutting-edge machine learning techniques, such as large-scale pre-trained models and KAN, into the field of GCI prediction, enhancing the accuracy and reliability of the predictions.
- Through experimental evaluations, we have assessed the effectiveness of large molecular models in GCI prediction tasks and explored the synergistic effects of their integration with other deep learning models, providing a reference for leveraging large molecular models to address GCI prediction problems.

## Results

### Datasets

In this study, we used a rigorously constructed dataset, namely GPCR-compound interaction dataset (GCIset) [30], which contains 356 GPCR entries and 5359 compound entries, represented in FASTA and SMILES formats, respectively, as detailed in Table 1. GPCR-compound interactions formed 15,343 pairs, with 7989 positive sample pairs (interactions) and 7354 negative sample pairs (non-interactions). For model evaluation, 1537 pairs were randomly selected as the test set, while 20% of the training set samples were randomly chosen to construct the validation set. It should be emphasized that this dataset was constructed following two rules: (i) collecting GCI data from experimentally validated databases; (ii) each ligand should exist in both classes simultaneously.

**Table 1:** Dataset statistics

Dataset	Compound	GPCRs	Positive	Negative
GCIset	5359	356	7989	7354
Train set	5031	333	5789	5255
Validation set	2132	253	1448	1314
Test set	1050	212	752	785

### Performance comparison with existing models

In this study, we evaluated the proposed EnGCI with four state-of-the-art models for predicting GPCR-Compound interactions, namely GraphDTA, MGraphDTA, TransformerCPI, and MFD-GDrug. Each of these models leverages different approaches for the task:

1. GraphDTA uses graph neural networks to predict the affinity between drugs and targets. The model represents drugs as molecular graphs and uses graph neural networks to learn the graph representation of drugs, while proteins are represented through one-hot encoding and 1D convolutional networks. Finally, the representation vectors of drugs and proteins are concatenated and the affinity score is predicted through a fully connected layer. In order to meet the requirements of binary classification tasks, the final layer of the GraphDTA adopts the sigmoid function, and the loss function is adjusted to binary cross-entropy (BCE), which is more suitable for binary classification.
2. MGraphDTA is a multi-scale graph neural network designed to predict drug-target interactions (DTIs) based on chemical structural features. For fair comparison, the original configuration of the model was retained and evaluated on the same GPCR-compound dataset.
3. TransformerCPI employs a transformer architecture with a self-attention mechanism to capture the interactions between compounds and protein sequences. The TransformerCPI model is a neural network based on the Transformer architecture, where the encoder is responsible for processing protein sequences and transforming them into sequence representations. The decoder processes the sequence of compound atoms and interacts with the output of the encoder to learn interactive features.
4. MFD-GDrug is a multimodal deep learning model. For proteins, the model integrates features extracted from ESM pretrained model and sequence information extracted using CNN. For drugs, the model integrates structural features extracted from Mol2vec and topological information of drug graph structures extracted through graph convolutional neural networks.

Table 2 summarizes the performance comparison of different models on the GCIset. In all key performance indicators, including AUC, PRC, precision, and recall, the model proposed in this study has demonstrated significant advantages, outperforming other comparative models. It is noteworthy that models such as GraphDTA, MGraphDTA, and TransformerCPI do not utilize the information from the large molecular model, and therefore, they are inferior to MFD-GDrug and EnGCI, which leverage the large molecular model information, in terms of the comprehensive performance indicators AUC and PRC. Although MFD-GDrug leads in overall performance, its recall rate is not as high as that of TransformerCPI. Both MFD-GDrug and EnGCI employ the information from the large molecular model, but they differ significantly in their model architectures. MFD-GDrug integrates the large molecular model information during the feature learning stage, achieving feature-level fusion. In contrast, the EnGCI model proposed in this paper constructs a classifier based solely on the large molecular model information and performs decision-level fusion. Benefiting from the progressiveness of model architecture, EnGCI significantly surpasses existing models in the four key indicators of AUC, PRC, precision, and recall, with performance improvements of approximately 2.89%, 1.83%, 3.96%, and 8.19% over MFD-GDrug, respectively. These results reflect the advanced nature and application potential of the EnGCI model in addressing the task of GPCR-compound interaction recognition.

**Table 2:** The comparison results of different models on GCIset, with bold values indicating the best performance among all competitive methods

Baseline	AUC	PRC	Precision	Recall
GraphDTA	0.80153	0.78246	0.75234	0.70251
MGraphDTA	0.84219	0.83757	0.76245	0.73156
TransformerCPI	0.86135	0.85291	0.77753	0.81254
MFD-GDrug	0.86294	0.86147	0.79125	0.75219
EnGCI	**0.88789**	**0.87732**	**0.82259**	**0.81377**

### Ablation experiment

To systematically evaluate the contribution of each module to the overall performance of the integrated model, we conducted exhaustive ablation experiments. In these experiments, we excluded various feature extraction and representation learning submodules, such as ESM, Uni-Mol, the GIN network, ResNet, 1D-CNN, and the KAN network, to analyze the impact of each submodules on the model’s performance. The experimental results are presented in Table 3.

**Table 3:** Results of ablation experiments on GCIset, where a “✓” indicates the inclusion of a module, a “✕” indicates its exclusion, and bold values represent the best performance among all competitive methods

Experiment	ESM	Uni-Mol	GIN	ResNet	CNN	KAN	AUC	PRC	Precision	Recall
I	✕	✓	✓	✓	✓	✓	0.86041	0.85519	0.76234	0.80129
II	✓	✕	✓	✓	✓	✓	0.85834	0.85012	0.77291	0.79235
III	✕	✕	✓	✓	✓	✓	0.80219	0.78341	0.72139	0.74135
IV	✓	✓	✓	✕	✓	✓	0.84132	0.82478	0.73845	0.78023
V	✓	✓	✕	✓	✓	✓	0.80234	0.80312	0.71548	0.77215
VI	✓	✓	✓	✓	✕	✓	0.85326	0.84321	0.76548	0.80253
VII	✓	✓	✓	✓	✓	✕	0.85139	0.83291	0.74526	0.77382
VIII	✕	✕	✓	✕	✓	✓	0.85737	0.83486	0.76973	0.80452
IX	✓	✓	✕	✓	✕	✓	0.81829	0.80604	0.71767	0.73078
X	✓	✓	✓	✓	✓	✓	**0.88789**	**0.87732**	**0.82259**	**0.81377**

In experiments I through III, we examined the effect of removing ESM and Uni-Mol, respectively. The results showed that when ESM was removed (experiment I), the AUC decreased to 0.86041, and the PRC dropped to 0.85519, indicating reductions of approximately 2.7% and 2.0%, respectively, compared to the baseline. When Uni-Mol was removed (experiment II), the AUC decreased to 0.85834, and the PRC dropped to 0.85012, with reductions of about 3.0% and 2.5%, respectively. In experiment III, where both pre-training models were removed simultaneously, the AUC dropped to 0.80219 and the PRC fell to 0.78341, resulting in a total decrease of approximately 9.6% in AUC and 10.8% in PRC. These results underscore the essential role of ESM and Uni-Mol in capturing advanced molecular features for effective GCI prediction.

In experiment IV, the residual-based 1D-CNN network used for feature extraction of both GPCR sequences and compound molecules was removed. The results showed that this removal led to a decrease in model performance, with the AUC decreasing to 0.84132 and the PRC to 0.82478, reflecting reductions of approximately 5.2% and 5.9%, respectively, compared to the baseline. Precision and recall also dropped to 0.73845 and 0.78023, highlighting the importance of the residual-based 1D-CNN network in effectively capturing features from both GPCR sequences and compound molecules.

In experiments V through VI, we analyzed the impact of removing the GIN for compound molecular structure extraction and the 1D-CNN for GPCR sequence feature extraction. The results show that when the GIN was removed (experiment V), the AUC and PRC dropped to 0.80234 and 0.80312, respectively, indicating decreases of approximately 9.6% and 8.5% compared to the baseline. Precision and recall also decreased to 0.71548 and 0.77215, highlighting the importance of the GIN in capturing structural information from compound molecular graphs. When the 1D-CNN was removed (experiment VI), the AUC decreased to 0.85326 and the PRC dropped to 0.84321, with reductions of about 3.9% and 4.2%, respectively. Precision and recall saw minor reductions, indicating that the 1D-CNN also contributes meaningfully to GPCR sequence feature extraction.

In experiment VII, we assessed the effect of removing the KAN from the model architecture. The results show that removing the KAN network led to a decrease in model performance, with the AUC dropping to 0.85139 and the PRC to 0.83291, representing reductions of approximately 4.1% and 5.0%, respectively, compared to the baseline. Precision and recall also declined to 0.74526 and 0.77382. These results highlight the important role of the KAN network in enhancing prediction accuracy and maintaining robust performance in GCI prediction tasks.

In experiments VIII and IX, we evaluated the performance of two individual models to assess their standalone contributions to the prediction task. For experiment VIII, where individual MSBM was tested, the AUC and PRC reached 0.85779 and 0.84346, respectively. Precision was 0.76977, and recall was 0.80452. Although the performance of MSBM is lower compared to the integrated model, it is still evident that MSBM can effectively extract the features of GPCRs and compounds for GCI prediction. In experiment IX, individual LMMBM was tested, resulting in an AUC of 0.81829 and a PRC of 0.80604. Precision and recall also dropped to 0.71767 and 0.73078, respectively. From experiments VIII and IX, it can be seen that the contribution of LMMBM is not as good as that of MSBM, because LMMBM is constructed upon two frozen large molecular models, which were initially trained through self-supervision and not specifically designed for GCI prediction, thereby inherently limiting the model’s discriminative capacity.

In experiment X, the results showed that the integrated model, combining ESM, Uni-Mol, the GIN network, ResNet, 1D-CNN, and the KAN network, achieved the highest performance in the GCI task. Compared to other models lacking essential components, the complete model has improved the AUC, PRC, precision, and recall by approximately $3.19\%^{\sim }10.68\%$, $2.59\%^{\sim }11.99\%$, $6.43\%^{\sim }14.97\%$, and $1.15\%^{\sim }10.40\%$, respectively. These results indicate that each module plays a significant role in enhancing the model’s performance, collectively providing a robust framework for addressing the complex GCI task.

### Comparative analysis of KAN and MLP as output layers

**Fig. 1:**
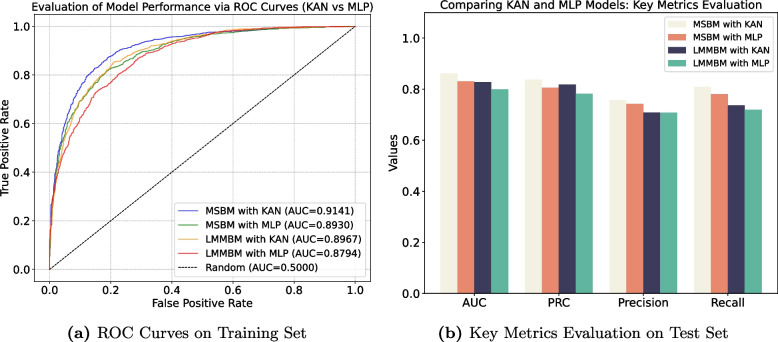
Comparison of model performance: MLP vs KAN

The performance of KAN and MLP as output layers was thoroughly evaluated, with the results presented in Fig. 1a and b. In Fig. 1a, ROC curve analysis highlights the superior performance of KAN over MLP. The MSBM with KAN achieves the highest AUC of 0.9141, followed by the LMMBM with KAN (AUC of 0.8967). In contrast, the MSBM with MLP get 0.8930 and the LMMBM with MLP get 0.8794. This suggests that KAN-based output layers provide better classification accuracy.

In Fig. 1b, the comparative evaluation on the test set reveals that KAN outperforms MLP across all key metrics-AUC, PRC, precision, and recall. The MSBM with KAN excels across all metrics, showing the highest values in each category. The LMMBM with KAN also demonstrates strong performance, closely following the MSBM. The MLP-based models lag slightly behind, particularly in precision and recall. These results demonstrate that KAN as an output layer enhances model performance.

### Model preference and feature importance in the integrated ensemble

In this study, we explored the preference of the MLP component within the integrated ensemble model for the outputs of the two modules: MSBM and LMMBM. Our experimental results suggest that the MLP exhibits a stronger affinity for the MSBM outputs over the LMMBM outputs. This is evidenced by the higher classification accuracy of the MSBM across both the training and test sets. To quantify this preference, we analyzed the first-layer weights of the MLP, which directly determine the influence of each model’s prediction on the final decision.

The calculation of these weights was done by extracting the first-layer weight matrix of the MLP and computing the average absolute weight for each input feature. Specifically, the weight matrix $W_{1}$ is given by:

1 $$\begin{aligned} W_{1}= \left[ \begin{array}{cc} w_{1,1} & w_{1,2}\\ w_{2,1} & w_{2,2}\\ \cdots & \cdots \\ w_{n,1} & w_{n,2} \end{array}\right] \end{aligned}$$

where $w_{j,1}$ refers to the weight between the $j^{th}$ neuron and the first input feature and $w_{j,2}$ refers to the weight between the $j^{th}$ neuron and the second input feature.

When calculating the average absolute weight for each input feature, the formula is applied separately to each feature. For input feature 1 (the output from MSBM), the absolute weights across all n neurons are summed, and similarly, for input feature 2 (the output from LMMBM), the absolute weights across all *n* neurons are summed. Specifically, the average absolute weight for input feature *i* is computed as:

2 $$\begin{aligned} w_{average}=\frac{1}{n} \sum \limits _{j=1}^{n}\left| w_{j,i} \right| \end{aligned}$$

where $w_{i,j}$ represents the weight between the $j^{th}$ neuron and the $i^{th}$ input feature.

The results showed that the weights assigned to the MSBM outputs (0.0312) were consistently larger than those assigned to the LMMBM outputs (0.0161), indicating that the MLP places greater importance on the MSBM predictions. This observation was further validated by the ablation experiments and results presented in Figs. 3 and 4, which demonstrate the enhanced predictive power of the MSBM component.

**Fig. 2:**
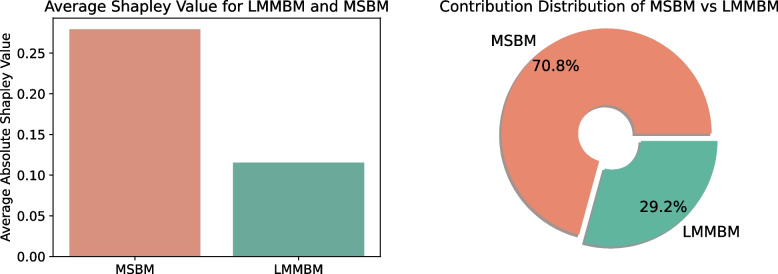
Shapley value comparison of MSBM and LMMBM

We employed SHAP (SHapley Additive exPlanations) analysis to gain a more comprehensive and robust understanding of how the two sub-models contribute to the final decision [41]. Shapley values are particularly useful for elucidating the contribution of each input feature. For the test set, the average absolute Shapley value of the MSBM output was determined to be 0.2792, while that of LMMBM was 0.1154. As depicted in Fig. 2, the bar chart on the left compares the average absolute Shapley values of MSBM and LMMBM. It is evident that MSBM has a substantially higher contribution to the final model decision. The pie chart on the right further demonstrates that MSBM contributes 70.8% to the decision-making process, compared to LMMBM’s 29.2%. These results are consistent with the previous MLP weight analysis, where the MSBM output exerted a greater influence on the model’s decision. The Shapley values provide a deeper and more detailed perspective on the feature importance of each model’s output within the ensemble.

Additionally, to further investigate the feature importance within the models, we conducted a series of feature ablation experiments. These experiments aimed to determine whether the models rely more on GPCR sequence features or compound features in their decision-making processes. The results of these ablation experiments are summarized in Tables 4 and 5.

**Table 4:** MSBM ablation experiment comparison

Experimental condition	AUC	PRC	Precision	Recall
Full feature set	0.85927	0.82314	0.78114	0.79632
GPCR removed	0.83192	0.78693	0.77217	0.77528
Compound removed	0.70135	0.68451	0.67429	0.71643

**Table 5:** LMMBM ablation experiment comparison

Experimental condition	AUC	PRC	Precision	Recall
Full feature set	0.81883	0.80509	0.72037	0.73408
GPCR removed	0.78625	0.76937	0.69495	0.71213
Compound removed	0.77732	0.75998	0.68896	0.70784

As shown in Table 4, the ablation experiments revealed that removing GPCR sequence features resulted in a minor performance decline, with the AUC decreasing by about 0.03 and the PRC decreasing by about 0.04. This suggests that while GPCR sequence features contribute to the model’s predictions, their impact is relatively limited. In contrast, removing compound features led to a more significant performance drop, with the AUC decreasing by about 0.16 and the PRC decreasing by about 0.14. These results highlight the model’s stronger reliance on compound-related information for accurate predictions.

As shown in Table 5, the ablation experiments revealed that removing GPCR sequence features resulted in a minor performance drop, with the AUC decreasing by about 0.03 and the PRC decreasing by about 0.04. This suggests that while GPCR sequence features contribute to the model’s predictions, their impact is relatively limited. In contrast, removing compound features led to a further decline in performance, with the AUC decreasing by about 0.04 and the PRC decreasing by about 0.05. These results further support the idea that compound-related information plays a crucial role in the model’s decision-making.

The results from the ablation experiments indicate that both models—MSBM and LMMBM—rely more heavily on compound features than GPCR sequence features. Specifically, the MSBM, which integrates molecular structure data such as atomic nodes, bond connections, topological information, and physicochemical properties, benefits from the rich, high-dimensional representation of compound-related information. These structural and chemical attributes are critical for the model’s predictive performance.

On the other hand, the LMMBM, while also showing a preference for compound characteristics, still incorporates GPCR sequence features. The sequence-based features in the LMMBM, processed as one-dimensional embeddings through the pre-trained ESM2 model, provide a more limited contribution compared to the compound features. The slightly lower reliance on GPCR sequence features in LMMBM may stem from their relatively conserved nature, which results in less discriminative power in certain cases, particularly when compared to the rich structural and chemical information inherent in compound features.

These findings underline the significance of compound-related information in the integrated model’s decision-making process, while GPCR sequence features play a secondary, yet supportive, role. Our study contributes valuable insights into the feature dependencies of integrated models in the context of GPCR-compound interactions, providing a foundation for future investigations aimed at improving model interpretability and enhancing biological research applications.

### Visualization analysis

In this study, we used the t-distributed stochastic neighbor embedding (t-SNE) [42] method to reduce the high-dimensional feature representations during model training to a two-dimensional space and visualize them to explore the feature distributions at different training stages.

**Fig. 3:**
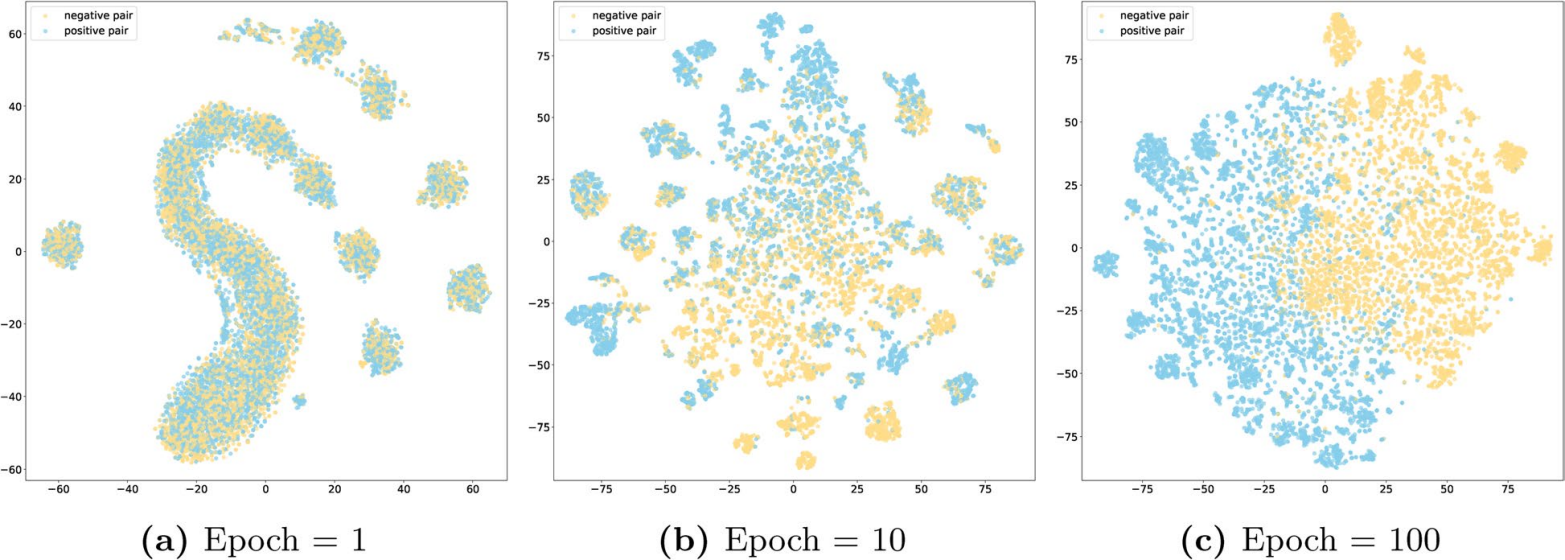
T-SNE visualization of learned representations in MSBM

Figure 3 shows the t-SNE results of MSBM under different training epochs. At epoch 1 (Fig. 3a), the sample distribution is more random, with no clear distinction between positive and negative samples, indicating that the model has not yet learned distinguishable features. By epoch 10 (Fig. 3b), the model begins to separate positive and negative samples, showing an emerging clustering trend in the feature space. By epoch 100 (Fig. 3c), clear clustering of positive and negative samples is visible, indicating the model’s high differentiation ability.

**Fig. 4:**
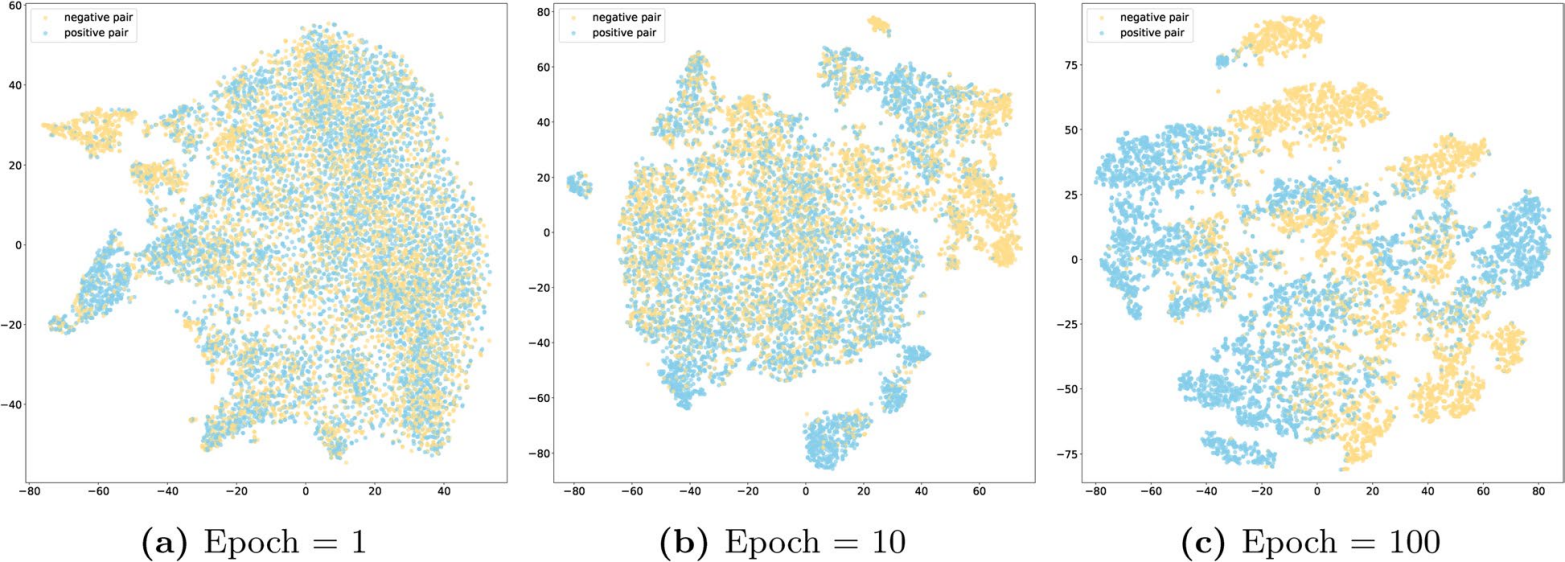
T-SNE visualization of learned representations in LMMBM

Similarly, Fig. 4 shows the feature distribution of LMMBM at different training stages. At epoch 1 (Fig. 4a), the samples are randomly distributed, with no significant feature information captured. By epoch 10 (Fig. 4b), the model starts recognizing differences between positive and negative samples, showing a tendency toward classification. By epoch 100 (Fig. 4c), the classification is much clearer, demonstrating the model’s strong ability to differentiate the samples.

**Fig. 5:**
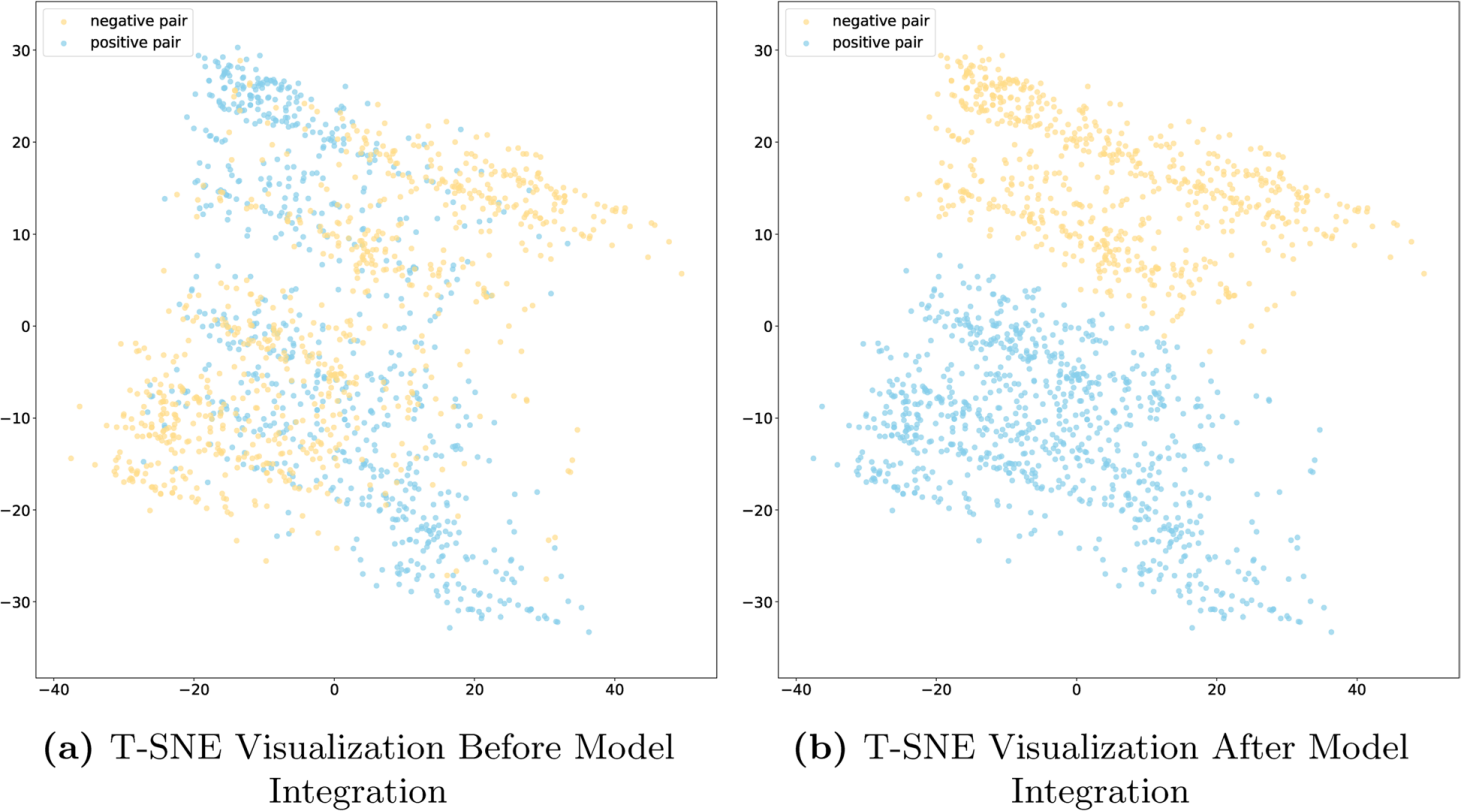
T-SNE comparison before and after integration into the model

Figure 5 presents a visual comparison of the feature distributions before and after applying the integrated model. Figure 5a illustrates the distribution of the intermediate layer dimensions of the output features extracted from MSBM and LMMBM before they are passed into the MLP. At this stage, there is still a considerable overlap between positive and negative samples, which complicates the classification task. To generate the visualization results in Fig. 5b, the following steps were employed: First, the 512-dimensional output from the last intermediate layer of both MSBM and LMMBM was extracted. These two feature representations were then concatenated to form a combined representation. This concatenated feature set was passed through the final MLP, where parameter training and learning occurred. The 256-dimensional intermediate layer output from the MLP was selected for visualization. To better visualize the data in a lower-dimensional space, T-SNE was applied to project this 256-dimensional representation into 2D, as shown in Fig. 5b. After undergoing this mapping process, a more distinct and unequivocal boundary is formed between positive and negative samples, as illustrated in Fig. 5b. This outcome indicates that by introducing MLP to integrate the information from the two models, we can significantly enhance the ability to differentiate between positive and negative samples, thereby further improving the accuracy and efficiency of classification.

Furthermore, it is clearly evident from the visualization results that the features learned by MSBM exhibit superior discriminative effectiveness between positive and negative samples compared to LMMBM. This is likely due to the fact that MSBM was trained from scratch, thus demonstrating better adaptability to the problem of GCI prediction. In contrast, LMMBM is constructed based on molecular representation vectors outputted by two frozen large molecular models. We are aware that these large molecular models were not originally designed for GCI prediction, and therefore, their discriminative capacity is somewhat limited. We have reason to speculate that if these two large molecular models can be fine-tuned, their discriminative ability may be further enhanced. However, it is worth noting that there is a good complementary effect between MSBM and LMMBM, as the integrated model demonstrates even greater strength in discriminative ability compared to either MSBM or LMMBM alone.

## Discussion

Our study presents the EnGCI model, which integrates two distinct modules for predicting GPCR-compound interactions (GCI). The results demonstrate that EnGCI outperforms existing state-of-the-art models, achieving an AUC of approximately 0.89 on a rigorously constructed GCI dataset. This significant improvement can be attributed to the synergistic effects of our dual-module approach, which leverages both deep learning and large molecular models to capture complex structural and functional information of molecules.

Our ablation study reveals the importance of each component within EnGCI. The removal of either ESM or Uni-Mol leads to a notable decrease in performance, highlighting the value of large molecular models in capturing nuanced molecular features. The KAN network also plays a pivotal role, offering a more expressive and efficient mechanism for integrating these features compared to traditional MLPs. In the visualization analysis, we found that the features learned by MSBM exhibit better discrimination between positive and negative samples, which may be due to MSBM being trained from scratch and thus having better adaptability to the GCI problem. Although LMMBM is based on two frozen large molecular models, their original design was not directly targeted at GCI prediction, resulting in certain limitations in their discrimination ability. However, through the integration strategy, the two models form a good complementary effect, making the integrated model demonstrate even stronger discrimination ability.

The EnGCI model proposed in this paper consists of two core components: the molecular structure-based module (MSBM) and the large molecular models-based module (LMMBM). The MSBM primarily focuses on extracting molecular structural features, for which we employ a combined architecture of GIN and 1D-CNN. This design choice stems from GIN’s exceptional performance in molecular graph structure representation learning and 1D-CNN’s advantage in capturing local structural patterns. The synergistic effect of these two effectively captures the key structural features of molecules. The LMMBM utilizes two pre-trained large molecular models to extract molecular information. Considering that the large molecular model is not specifically optimized for the GCI prediction task, we introduce a 1D-CNN layer after it to achieve feature adaptation and task-specific information extraction. This design significantly reduces the number of parameters while ensuring model performance. Notably, at the end of both modules, we employ KAN for decision-making, primarily due to KAN’s ability to achieve excellent predictive performance with a more streamlined parameter scale. Finally, we design a MLP to integrate the outputs of the MSBM and LMMBM modules, achieving the final predictive decision by automatically learning the complex relationships between the modules. This architectural design ensures model performance while fully considering the model’s efficiency.

While EnGCI shows promise, there are areas for future enhancement. Firstly, the generalizability of our model could be further validated with a more diverse and extensive dataset. Secondly, the interpretability of EnGCI is an area that requires attention. Understanding the decision-making process and identifying key features that influence GCI predictions is very important for translating our model’s predictions into actionable insights in drug discovery.

## Conclusions

The present study introduces EnGCI, a novel and integrated framework designed to enhance the prediction of GPCR-compound interactions. Our model stands out due to its innovative dual-module architecture that synergistically combines the strengths of graph isomorphism networks, convolutional neural networks, and large-scale pre-trained molecular models with the Kolmogorov-Arnold network for decision-making.The EnGCI model has demonstrated superior performance over existing benchmarks, which underscores its potential in the realm of drug discovery and chemogenomics.

The EnGCI model’s robust predictive capabilities have significant implications for GPCR drug discovery. By accurately predicting which compounds are likely to interact with specific GPCRs, we can streamline the drug development process, reducing the time and cost associated with experimental testing. Furthermore, EnGCI’s ability to handle large-scale predictions may facilitate high-throughput screening efforts.

## Methods

### Overview

**Fig. 6:**
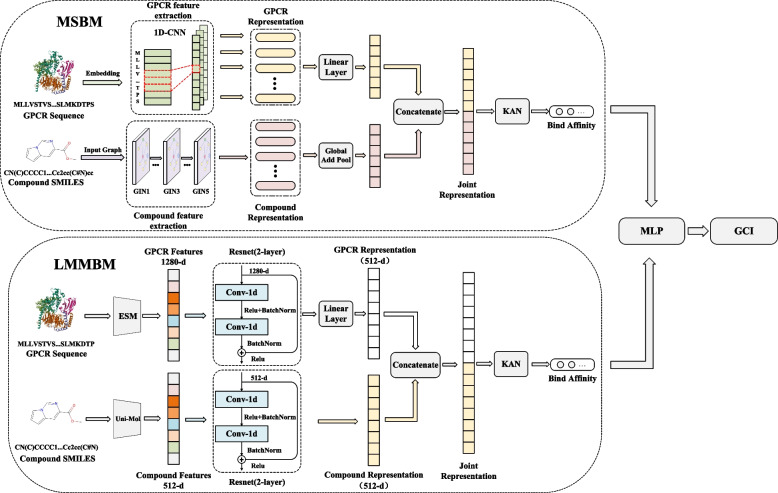
EnGCI model structure

EnGCI predicts GCI by combining multimodal features derived from compound molecules and GPCR sequences. The workflow of EnGCI consists of two main models, as shown in Fig. 6.

MSBM: For compound molecules, we first convert SMILES sequences into graphical structures, then extract molecular features using the graph isomorphism network (GIN). For GPCR sequences, we use a 1D convolutional neural network (1D-CNN) for feature extraction. Finally, the prediction results are output through the KAN.

LMMBM: We introduce the Uni-Mol pre-trained model to extract high-level features of compounds, generating a 512-dimensional feature representation, which is then further extracted using a residual-based 1D-CNN network. For GPCR sequences, an ESM pretrained model was used to generate 1280-dimensional feature representations, and additional feature extraction was performed by a residual-based 1D-CNN network. Then use KAN for classification.

Finally, we integrate the predictions of the two models dynamically via a MLP and evaluate the performance of the integrated model on a test set.

### MSBM representation learning

Currently, deep learning models based on CNNs are widely used for feature extraction from GPCR sequences [43]. 1D-CNNs can quickly capture local contextual features and short-range dependencies in GPCR sequences through sliding convolutional operations. In MSBM, we leverage 1D-CNNs to extract local features from GPCR sequences. First, amino acids in a GPCR sequence are converted to integer-encoded sequences. For example, alanine (A) is encoded as integer 1, aspartic acid (D) as integer 3, glutamic acid (E) as integer 4, cysteine (C) as integer 5, and so on. To fit the model’s input requirements, GPCR sequences are populated or trimmed to a fixed residue sequence length of S. If sequences are shorter than S, zero padding is used to maintain consistent length. These integer-encoded sequences are converted to d-dimensional vector representations through the embedding layer, forming a feature matrix for each GPCR sequence. This matrix is then inputted into the 1D-CNN for local feature extraction. Through convolutional operations, the 1D-CNN extracts increasingly abstract features layer by layer for subsequent prediction tasks.

3 $$\begin{aligned} H_{i}=\sigma \left( \sum \limits _{k=0}^{K-1} W_{k} \cdot x_{i+k} +b \right) \end{aligned}$$

where $H_{i}$ represents the output feature at position *i* in the GPCR sequence after the convolution operation. $W_{k}$ is the weight at the *k*th position of the convolution kernel, and $x_{i+k}$ denotes the feature vector at position $i+k$ in the input sequence. Here, *K* is the size of the convolution kernel, *b* is the bias term, and $\sigma \left( \cdot \right)$ is the nonlinear activation function, with $ReLU\left( \cdot \right)$ applied in our experiments. To achieve effective integration with the features of compound molecules, the sequence feature matrix of GPCR derived from a one-dimensional convolutional neural network (1D-CNN) is first flattened into a one-dimensional vector form, and then a linear transformation is applied to obtain the transformed feature vector $H_{G_{1}}$, ensuring its dimensionality aligns with the representation of compound molecules. This transformation strategy greatly facilitates the compatibility between the two molecular representation forms, thereby enhancing their joint representation capabilities and effects.

Pharmaceutical molecules typically exhibit complex two-dimensional (2D) or three-dimensional (3D) structures, and these structural features significantly influence their chemical properties and biological activities. However, such complex molecular structures are difficult to fully characterize using traditional linear description methods. To efficiently extract and characterize the structural information of compound molecules, we use a GIN in MSBM. First, we convert the SMILES sequence of a compound into a graph $G=(V,E)$, where *V* is the set of nodes representing the atoms in the compound molecule, and each node is represented by an *L*-dimensional feature vector. *E* is the set of edges representing the chemical bonds between atoms, and the adjacency matrix $A\in R^{N\times N }$ describes the connectivity between nodes. In the GIN model, through layer-by-layer convolution, the features of each node depend not only on itself but also aggregate features from neighboring nodes. The key feature of GIN is its additive aggregation mechanism, which allows the model to better capture relationships between nodes in the graph, and the local structural information of the molecule. After each convolution layer, the node features aggregate both their own features and the information of their neighboring nodes. Upon completing all convolution layers, we apply a global pooling operation (e.g., global add pooling) across all nodes to obtain a single feature vector $H_{C_{1}}$ that represents the entire molecule. This global feature vector $H_{C_{1}}$ captures both the local structural details and overall connectivity of the molecule, making it suitable for further integration with the GPCR feature vector in downstream tasks.

4 $$\begin{aligned} h_{v}^{\left( k+1 \right) } =MLP\left( \left( 1+\varepsilon \right) h_{v}^{\left( k \right) }+ {\sum \nolimits _{u\in N\left( v \right) }} h_{u}^{\left( k \right) } \right) \end{aligned}$$

In Eq. (4), $h_{v}^{\left( k \right) }$ denotes the feature matrix of node *v* at the *k*th layer, $N\left( v \right)$ denotes the set of neighboring nodes of node *v*, and $\varepsilon$ is a learnable parameter, controlling the proportion of contributions from the node’s own features and the neighboring features. MLP (multi-layer perceptron) is used for updating the node features through nonlinear transformations.

5 $$\begin{aligned} H_{C_{1}} = \sum \nolimits _{v\in G}h_{v}^{\left( k \right) } \end{aligned}$$

where $H_{C_{1}}$ is a graph-wide feature representation of the graph *G*, and ${\sum \nolimits _{V\in G}}$ denotes a summation operation on the feature vectors of all nodes *v* in the graph.

To integrate the feature information from the GPCR sequence and the compound molecule, we concatenated the extracted GPCR feature vector $H_{G_{1}}$ and the compound molecule vector $H_{C_{1}}$ to create a fused feature vector $H_{concat_{1}}$.

6 $$\begin{aligned} H_{concat_{1}}=\left[ H_{G_{1}}\left| \right| H_{C_{1}} \right] \end{aligned}$$

The feature vector $H_{concat_{1}}$ is directly passed into the KAN network for the binary prediction task. Unlike the traditional multilayer perceptron, the KAN network is based on the Kolmogorov-Arnold representation theorem, which expresses complex multidimensional functional relationships through a series of univariate function combinations [44].

7 $$\begin{aligned} {Z}' =\sum \limits _{q=1}^{2n+1} \Phi _{q}\left( \sum \limits _{p=1}^{n} \varphi _{q,p}\left( Z_{p} \right) \right) \end{aligned}$$

In Eq. (7), *q* denotes the index of the function in the range $1\le q\le 2n+1$, and *p* denotes the index of the input feature dimension in the range $1\le p\le n$, where *n* is the size of the feature dimension. $Z_{p}$ denotes the *p*th dimension of the fused feature vector. The function $\Phi _{q}$ represents the *q*th output univariate function, which acts on the result of all the *p*-dimensional features processed through the function. The function $\varphi _{q,p} \left( Z_{p} \right)$ represents the transformation of the input feature $Z_{p}$ by a univariate function that generates the result corresponding to *q*. Each *p*-dimensional feature is processed through a different univariate function $\varphi$, and the results are combined to form a complex multidimensional functional relationship.

KAN offers greater expressiveness and efficiency compared to traditional MLP, particularly in capturing complex nonlinear features in GPCR-compound interaction prediction (GCI), which effectively improves the accuracy and robustness of the model. The classification process is optimized using a binary cross-entropy (BCE) loss function with the following mathematical expression:

8 $$\begin{aligned} Loss & = -\frac{1}{N} \sum \limits _{i} \left[ y_{i} \cdot \log \left( p\left( y_{i} \right) \right) \right. \nonumber \\ & \quad + \left( 1-y_{i} \right) \cdot \log \left( 1-p\left( y_{i} \right) \right) \left. \right] \end{aligned}$$

In Eq. (8), $y_{i}$ denotes the true label of the interaction (whether an interaction exists or not), and $p\left( y_{i} \right)$ is the predicted probability of an interaction between a compound and a GPCR. *N* represents the sample size, and log denotes the natural logarithm (ln).

Finally, we optimized all learnable parameters of MSBM using backpropagation to minimize the designated loss function, yielding the prediction output, $Y_{1}$.

### LMMBM representation learning

In the LMMBM, GPCR sequence feature extraction utilizes ESM2 [45], specifically the esm2_t33_650M_UR50D version, a pre-trained self-supervised model designed to capture global representations and biological information within protein sequences. ESM2 employs a technique similar to masked language modeling (MLM), where the model learns complex syntactic and semantic relationships by predicting masked amino acids within sequences. This process enables ESM2 to capture long-range dependencies, akin to human language models, and identify remote interactions and global structural patterns that are crucial for GPCR folding and domain interactions.

The self-attention mechanism in ESM2 allows it to effectively capture long-distance interactions within protein sequences, generating a 1280-dimensional global representation. This global representation is produced through a pooling operation, where information across all nodes in the sequence is aggregated based on the node number dimension. The pooling process ensures that the final 1280-dimensional vector encapsulates both local and global features, representing functional, structural, and evolutionary patterns. This vector is enriched with critical information, including active sites, functional domains, and 3D structural details, providing a comprehensive feature set for downstream tasks. By highlighting both conserved and novel functional regions across diverse species, this global representation enables more accurate predictions for GPCR-related applications.

After the ESM2 model generates the feature representation, further feature processing is done by the residual-based 1D-CNN network. The introduction of ResNet addresses the common problem of gradient vanishing in deep neural networks, ensuring smooth information flow via residual connectivity. This approach allows for the effective transfer of features, even as the network depth increases. The multi-layer convolutional operations in ResNet help the model further extract local patterns and deeper structural features in GPCR sequences, particularly capturing key elements such as local functional domains, secondary structures, and mutation effects.

9 $$\begin{aligned} {H_{G_{2}} }' =\sigma \left( H_{G_{2}}+ Conv\left( H_{G_{2}} \right) \right) \end{aligned}$$

In Eq. (9), $H_{G_{2}}$ is the feature vector generated from the ESM model, and *Conv* denotes the convolution operation used to extract local patterns in the GPCR features. The skip connection sums the input $H_{G_{2}}$ and the result after convolution to prevent the vanishing gradient problem and enhance the expression of the features. When processing the features of GPCRs, the 1D-CNN maintains their feature dimensions unchanged. Subsequently, the output of the 1D-CNN passes through a linear layer to obtain a feature representation ${H_{G_{2}}}''$, which has dimensions that are consistent with those of the compound features.

To capture high-level features of compound molecules, we use the pre-trained Uni-Mol model [46], which integrates chemical symbolic representations, such as SMILES, with spatial configurations, enabling the capture of both topological and geometrical relationships within molecules for enriched feature representation. Uni-Mol’s training includes unsupervised tasks like masked atom prediction, which is similar to masked language modeling (MLM) in NLP, and atom pair distance prediction. These tasks help the model learn both local atomic relationships and global 3D geometry.

The 512-dimensional features output from Uni-Mol are obtained using the “CLS” token, which extracts a compact representation of the compound sequence. These features are further refined by a residual network (ResNet) to improve representation quality. Through multilayer convolutional operations, ResNet extracts complex nonlinear relationships, which are crucial for compound characterization, as chemical properties and biological activities often follow nonlinear patterns. This step refines Uni-Mol’s molecular features, allowing a more precise capture of intricate patterns in compound molecules.

10 $$\begin{aligned} {H_{C_{2}} }' =\sigma \left( H_{C_{2}}+ Conv\left( H_{C_{2}} \right) \right) \end{aligned}$$

where $H_{C_{2}}$ represents the feature vector generated by the Uni-Mol model, while *Conv* denotes the convolution operation applied to extract local patterns. The activation function $\sigma$ is applied to the sum of $H_{C_{2}}$ and its convolutional transformation, resulting in the enhanced feature representation ${H_{C_{2}} }'$.

11 $$\begin{aligned} H_{concat_{2}}=\left[ {H_{G_{2}}}''\left| \right| {H_{C_{2}} }' \right] \end{aligned}$$

Same as in MSBM, we concatenate the extracted GPCR feature vector ${H_{G_{2}}}''$ and the compound feature vector ${H_{C_{2}} }'$ to form a unified representation, denoted as $H_{concat_{2}}$. This concatenated vector is then input directly into the KAN for binary classification, predicting the likelihood of GPCR-compound interactions.

### Multimodal ensemble learning

The core innovation of this study focuses on a multimodal learning strategy, aiming to integrate diverse features derived from compounds and GPCRs to enhance the predictive performance of GCI. Specifically, MSBM employs both graph neural networks and convolutional neural networks, both of which demonstrate exceptional abilities in capturing local sequence features, whereas LMMBM relies on advanced large molecular models to deeply explore the structural properties and functional information of molecules. To fully integrate the complementary advantages of MSBM and LMMBM, we have introduced MLP as an information fusion module. In this framework, the predictive outputs Y1 and Y2 from MSBM and LMMBM are fed into the MLP. Leveraging its adaptive learning capabilities, the MLP precisely captures and integrates the feature correlations between the two models, thereby generating a more accurate final prediction $Y_{final}$, as detailed in Eq. (12).

12 $$\begin{aligned} Y_{final} =MLP\left( Y_{1},Y_{2} \right) \end{aligned}$$

### Training and validation

In MSBM, GPCR sequences are initially converted into integer sequences using one-hot encoding, followed by embedding through a one-dimensional convolutional layer (1D-CNN). This layer is configured with 128 input channels, 32 output channels, and a kernel size of 5, with padding set to 2 to maintain the original sequence length. The output from this layer is passed through a fully connected layer, yielding a 128-dimensional feature representation of the GPCR sequence. For compound molecular structures, MSBM utilizes a graph isomorphism network (GIN) to perform feature extraction. Five sequential GIN layers are employed, each with a hidden dimension of 32. Each layer comprises two linear transformations with ReLU activation and batch normalization to enhance training stability and improve convergence. The graph-based features of the compound molecule are subsequently aggregated using global addition pooling, resulting in a single vector representation. This representation is then processed through a fully connected layer, producing a 128-dimensional feature vector for the compound molecule. The resulting GPCR and compound feature vectors are concatenated and input into the KAN for classification. The KAN is configured with an input dimension of 1024, a hidden layer of 256 dimensions, and an output dimension of 1, offering the likelihood of GCI.

In LMMBM, GPCR sequences are embedded using the pretrained ESM model, yielding a 1280-dimensional global feature representation, while compound molecules are embedded using the Uni-Mol model, resulting in a 512-dimensional feature vector. Both GPCR and compound features are then processed through two ResNet blocks for each, with each block comprising two convolutional layers with kernel sizes of 3 for both compounds and GPCR features. These blocks include skip connections to mitigate potential vanishing gradient issues, with batch normalization, ReLU activation, and a dropout rate of 0.5 to enhance stability and generalization. After ResNet processing, the GPCR feature dimensionality is reduced using a linear layer that maps the 1280-dimensional GPCR feature vector to 512 dimensions, aligning it with the compound feature vector for seamless integration. The combined feature vectors, including compound features and GPCR features, are input into KAN for binary classification, where the hyperparameter configuration of KAN is consistent with that of MSBM.

The MSBM and LMMBM were implemented using the PyTorch framework and trained on a Nvidia GeForce RTX 3090 GPU. The training was performed using 80% of the dataset for model learning and 20% for validation, with a total of 1000 rounds of training. For the binary classification task of both models, multiple evaluation metrics were used to comprehensively assess performance, including area under receiver operating characteristic curve (AUC), area under precision recall curve (PRC), precision, and recall. AUC reflects the classification performance across different thresholds and can be used as a criterion for early stopping during training. PRC focuses on the model’s performance across different precision and recall rates and is particularly suitable for handling class imbalance. It sensitively captures changes in performance. Precision indicates the proportion of predicted positive samples that are truly positive, while recall measures the proportion of actual positive samples correctly identified by the model. These metrics complement each other, providing a comprehensive evaluation of the model’s classification performance and helping to analyze and optimize the model for different tasks.

In the validation phase, we analyzed whether the prediction results from the two models on the validation set complement each other. The outputs of the two models were integrated via a multilayer perceptron (MLP), and the performance of the integrated model was evaluated on the test set.

## Data Availability

All data generated or analyzed during this study are included in this published article and publicly available repositories. The source codes and data have been deposited in GitHub under the following link: https://github.com/lwhaoooo/EnGCI and on Figshare with the DOI: https://doi.org/10.6084/m9.figshare.28690157.v3.

## Abbreviations

MSBM: Molecular structure-based module
LMMBM: Large molecular models-based module
GCI: GPCR-compound interaction
GIN: Graph isomorphism network
GCN: Graph convolutional network
GAT: Graph attention networks
1D-CNN: 1D convolutional neural network
KAN: Kolmogorov-Arnold network
MLP: Multi-layer perceptron
ResNet: Residual network
AUC: Area under receiver operating characteristic curve
PRC: Area under precision recall curve
BCE: Binary cross-entropy
t-SNE: t-distributed stochastic neighbor embedding

## References

[1] Weis WI, Kobilka BK. Structural insights into G-protein-coupled receptor activation. Curr Opin Struct Biol. 2008;18(6):734–40. 18957321 10.1016/j.sbi.2008.09.010 PMC4019673

[2] Thompson MD, Burnham WM, Cole DE. The G protein-coupled receptors: pharmacogenetics and disease. Crit Rev Clin Lab Sci. 2005;42(4):311–89. 16281738 10.1080/10408360591001895

[3] Lagerström MC, Schiöth HB. Structural diversity of G protein-coupled receptors and significance for drug discovery. Nat Rev Drug Discov. 2008;7(4):339–57. 18382464 10.1038/nrd2518

[4] Hauser AS, Attwood MM, Rask-Andersen M, Schiöth HB, Gloriam DE. Trends in GPCR drug discovery: new agents, targets and indications. Nat Rev Drug Discov. 2017;16(12):829–42. 29075003 10.1038/nrd.2017.178 PMC6882681

[5] Thomsen AR, Plouffe B, Cahill TJ, Shukla AK, Tarrasch JT, Dosey AM, et al. GPCR-G protein--arrestin super-complex mediates sustained G protein signaling. Cell. 2016;166(4):907–19. 10.1016/j.cell.2016.07.004 PMC5418658 27499021

[6] Rasmussen SG, DeVree BT, Zou Y, Kruse AC, Chung KY, Kobilka TS, et al. Crystal structure of the 2 adrenergic receptor-Gs protein complex. Nature. 2011;477(7366):549–55. 10.1038/nature10361 PMC3184188 21772288

[7] Venkatakrishnan A, Deupi X, Lebon G, Tate CG, Schertler GF, Babu MM. Molecular signatures of G-protein-coupled receptors. Nature. 2013;494(7436):185–94. 23407534 10.1038/nature11896

[8] Manglik A, Kruse AC. Structural basis for G protein-coupled receptor activation. Biochemistry. 2017;56(42):5628–34. 28967738 10.1021/acs.biochem.7b00747 PMC6613644

[9] Venkatakrishnan A, Ma AK, Fonseca R, Latorraca NR, Kelly B, Betz RM, et al. Diverse GPCRs exhibit conserved water networks for stabilization and activation. Proc Natl Acad Sci. 2019;116(8):3288–93. 30728297 10.1073/pnas.1809251116 PMC6386714

[10] Rosenbaum DM, Rasmussen SG, Kobilka BK. The structure and function of G-protein-coupled receptors. Nature. 2009;459(7245):356–63. 19458711 10.1038/nature08144 PMC3967846

[11] Jacobson KA. New paradigms in GPCR drug discovery. Biochem Pharmacol. 2015;98(4):541–55. 26265138 10.1016/j.bcp.2015.08.085 PMC4967540

[12] Flock T, Hauser AS, Lund N, Gloriam DE, Balaji S, Babu MM. Selectivity determinants of GPCR-G-protein binding. Nature. 2017;545(7654):317–22. 28489817 10.1038/nature22070 PMC5846738

[13] Shiraishi A, Niijima S, Brown J, Nakatsui M, Okuno Y. Chemical genomics approach for gpcr-ligand interaction prediction and extraction of ligand binding determinants. J Chem Inf Model. 2013;53(6):1253–62. 23721295 10.1021/ci300515z

[14] Nemoto W, Yamanishi Y, Limviphuvadh V, Saito A, Toh H. GGIP: structure and sequence-based GPCR-GPCR interaction pair predictor. Proteins Struct Funct Bioinformatics. 2016;84(9):1224–33. 10.1002/prot.25071 27191053

[15] Xiao X, Min JL, Wang P, Chou KC. iGPCR-Drug: a web server for predicting interaction between GPCRs and drugs in cellular networking. PLoS ONE. 2013;8(8):e72234. 24015221 10.1371/journal.pone.0072234 PMC3754978

[16] Zheng Y, Wu Z. A machine learning-based biological drug-target interaction prediction method for a tripartite heterogeneous network. ACS Omega. 2021;6(4):3037–45. 33553921 10.1021/acsomega.0c05377 PMC7860102

[17] Hu J, Li Y, Yang JY, Shen HB, Yu DJ. GPCR-drug interactions prediction using random forest with drug-association-matrix-based post-processing procedure. Comput Biol Chem. 2016;60:59–71. 26674225 10.1016/j.compbiolchem.2015.11.007

[18] Qiu W, Lv Z, Hong Y, Jia J, Xiao X. BOW-GBDT: a GBDT classifier combining with artificial neural network for identifying GPCR-drug interaction based on wordbook learning from sequences. Front Cell Dev Biol. 2021;8:623858. 33598456 10.3389/fcell.2020.623858 PMC7882597

[19] Karimi S, Ahmadi M, Goudarzi F, Ferdousi R. A computational model for GPCR-ligand interaction prediction. J Integr Bioinforma. 2021;18(2):155–65. 10.1515/jib-2019-0084 PMC7790179 34171942

[20] Wang P, Huang X, Qiu W, Xiao X. Identifying GPCR-drug interaction based on wordbook learning from sequences. BMC Bioinformatics. 2020;21:1–17. 32312232 10.1186/s12859-020-3488-8 PMC7171867

[21] Ye Q, Zhang X, Lin X. Drug-target interaction prediction via multiple classification strategies. BMC Bioinformatics. 2022;22(Suppl 12):461. 35057737 10.1186/s12859-021-04366-3 PMC8772044

[22] Redkar S, Mondal S, Joseph A, Hareesha K. A machine learning approach for drug-target interaction prediction using wrapper feature selection and class balancing. Mol Inform. 2020;39(5):1900062. 10.1002/minf.201900062 32003548

[23] Oh J, Ceong HT, Na D, Park C. A machine learning model for classifying G-protein-coupled receptors as agonists or antagonists. BMC Bioinformatics. 2022;23(Suppl 9):346. 35982407 10.1186/s12859-022-04877-7 PMC9389651

[24] Zeng X, Xiang H, Yu L, Wang J, Li K, Nussinov R, et al. Accurate prediction of molecular properties and drug targets using a self-supervised image representation learning framework. Nat Mach Intell. 2022;4(11):1004–16.

[25] Yamane H, Ishida T. Helix encoder: a compound-protein interaction prediction model specifically designed for class A GPCRs. Front Bioinforma. 2023;3:1193025. 10.3389/fbinf.2023.1193025 PMC10250622 37304403

[26] Wen M, Zhang Z, Niu S, Sha H, Yang R, Yun Y, et al. Deep-learning-based drug-target interaction prediction. J Proteome Res. 2017;16(4):1401–9. 28264154 10.1021/acs.jproteome.6b00618

[27] Lei Y, Li S, Liu Z, Wan F, Tian T, Li S, et al. A deep-learning framework for multi-level peptide-protein interaction prediction. Nat Commun. 2021;12(1):5465. 34526500 10.1038/s41467-021-25772-4 PMC8443569

[28] Wang YB, You ZH, Yang S, Yi HC, Chen ZH, Zheng K. A deep learning-based method for drug-target interaction prediction based on long short-term memory neural network. BMC Med Informat Decis Making. 2020;20:1–9. 10.1186/s12911-020-1052-0 PMC7079345 32183788

[29] Tian Z, Peng X, Fang H, Zhang W, Dai Q, Ye Y. MHADTI: predicting drug-target interactions via multiview heterogeneous information network embedding with hierarchical attention mechanisms. Brief Bioinforma. 2022;23(6):bbac434. 10.1093/bib/bbac434 36242566

[30] Chen L, Tan X, Wang D, Zhong F, Liu X, Yang T, et al. TransformerCPI: improving compound-protein interaction prediction by sequence-based deep learning with self-attention mechanism and label reversal experiments. Bioinformatics. 2020;36(16):4406–14. 32428219 10.1093/bioinformatics/btaa524

[31] Wang H, Zhou G, Liu S, Jiang JY, Wang W. Drug-target interaction prediction with graph attention networks. 2021. arXiv preprint arXiv:2107.06099.

[32] Yadav P, Mollaei P, Cao Z, Wang Y, Farimani AB. Prediction of GPCR activity using machine learning. Comput Struct Biotechnol J. 2022;20:2564–73. 35685352 10.1016/j.csbj.2022.05.016 PMC9163700

[33] Nguyen T, Le H, Quinn TP, Nguyen T, Le TD, Venkatesh S. GraphDTA: predicting drug-target binding affinity with graph neural networks. Bioinformatics. 2021;37(8):1140–7. 33119053 10.1093/bioinformatics/btaa921

[34] Zhang H, Fan H, Wang J, et al. Revolutionizing GPCR–ligand predictions: DeepGPCR with experimental validation for high-precision drug discovery[J]. Brief Bioinform. 2024;25(4):bbae281. 10.1093/bib/bbae281 PMC11167311 38864340

[35] Wang P, Huang X, Qiu W, et al. Identifying GPCR-drug interaction based on wordbook learning from sequences[J]. BMC bioinform. 2020;21:150. 10.1186/s12859-020-3488-8 PMC7171867 32312232

[36] Gan Y, Liu W, Xu G, Yan C, Zou G. DMFDDI: deep multimodal fusion for drug-drug interaction prediction. Brief Bioinforma. 2023;24(6):bbad397. 10.1093/bib/bbad397 37930025

[37] Gu X, Liu J, Yu Y, Xiao P, Ding Y. MFD-GDrug: multimodal feature fusion-based deep learning for GPCR-drug interaction prediction. Methods. 2024;223:75–82. 38286333 10.1016/j.ymeth.2024.01.017

[38] Chu SK, Narang K, Siegel JB. Protein stability prediction by fine-tuning a protein language model on a mega-scale dataset. PLOS Comput Biol. 2024;20(7):e1012248. 39038042 10.1371/journal.pcbi.1012248 PMC11293664

[39] Kurata H, Harun-Or-Roshid M, Tsukiyama S, Maeda K. PredIL13: stacking a variety of machine and deep learning methods with ESM-2 language model for identifying IL13-inducing peptides. PLoS ONE. 2024;19(8):e0309078. 39172871 10.1371/journal.pone.0309078 PMC11340954

[40] Bryant P, Kelkar A, Guljas A, Clementi C, Noé F. Structure prediction of protein-ligand complexes from sequence information with Umol. Nat Commun. 2024;15(1):4536. 38806453 10.1038/s41467-024-48837-6 PMC11133481

[41] Lundberg SM, Lee SI. A unified approach to interpreting model predictions. Adv Neural Inf Process Syst. 2017;30:4765–74.

[42] Van der Maaten L, Hinton G. Visualizing data using t-sne. J Mach Learn Res. 2008;9(86):2579–605.

[43] Lee I, Keum J, Nam H. DeepConv-DTI: prediction of drug-target interactions via deep learning with convolution on protein sequences. PLoS Comput Biol. 2019;15(6):e1007129. 31199797 10.1371/journal.pcbi.1007129 PMC6594651

[44] Liu Z, Wang Y, Vaidya S, Ruehle F, Halverson J, Soljačić M, et al. KAN: Kolmogorov-Arnold networks. 2024. arXiv preprint arXiv:2404.19756.

[45] Luo Z, Wang R, Sun Y, Liu J, Chen Z, Zhang YJ. Interpretable feature extraction and dimensionality reduction in ESM2 for protein localization prediction. Brief Bioinforma. 2024;25(2):bbad534. 10.1093/bib/bbad534 PMC10818170 38279650

[46] Zhou G. et al. Uni-Mol: a universal 3D molecular representation learning framework. Preprint at ChemRxiv. 2023. https://chemrxiv.org/engage/chemrxiv/article-details/6402990d37e01856dc1d1581.

